# Dual-energy X-ray absorptiometry-measured lean mass and clinical bone fracture in elderly Japanese men: the Fujiwara-kyo Osteoporosis Risk in Men (FORMEN) cohort study

**DOI:** 10.1265/ehpm.25-00449

**Published:** 2026-03-12

**Authors:** Katsuyasu Kouda, Yuki Fujita, Takahiro Tachiki, Akihiro Takada, Soulattana Vongsakit, Yuki Murakami, Junko Tamaki, Kumiko Ohara, Etsuko Kajita, Akemi Nitta, Kazuhiro Uenishi, Nozomi Okamoto, Masayuki Iki

**Affiliations:** 1 Department of Hygiene and Public Health, Faculty of Medicine, Kansai Medical University, 2-5-1 Shin-machi, Hirakata, Osaka 573-1010, Japan; 2 Department of Nursing, Faculty of Nursing, University of Kochi, 2751-1 Ike, Kochi, Kochi 781-8515, Japan; 3 Department of Physical Therapy, Faculty of Healthcare Sciences, Aino University, 4-5-4 Higashi Ota, Ibaraki, Osaka 567-0012, Japan; 4 Department of Hygiene and Public Health, Faculty of Medicine, Osaka Medical and Pharmaceutical University, 2-7 Daigakumachi, Takatsuki, Osaka 569-8686, Japan; 5 Department of Epidemiology for Community Health and Medicine, Kyoto Prefectural University of Medicine, 465 Kajii-cho, Kawaramachi-Hirokoji, Kamigyo, Kyoto, Kyoto 602-8566, Japan; 6 Nagoya University, Furo-cho, Chikusa-ku, Nagoya, Aichi 464-8601, Japan; 7 Laboratory of Physiological Nutrition, Kagawa Nutrition University, 3-9-21 Chiyoda, Sakado, Saitama 350-0288, Japan; 8 Graduate School of Education, Hyogo University of Teacher Education, 942-1 Shimokume, Kato, Hyogo 673-1494, Japan

**Keywords:** Body composition, Clinical bone fracture, Densitometry, Epidemiology, Lean mass

## Abstract

**Background:**

Bone fractures pose a significant socioeconomic burden for both women and men in aging societies. It has been reported that lean mass (LM) measured by dual-energy X-ray absorptiometry (DXA) may be associated with fractures. However, evidence regarding the relationship between DXA-measured LM and fractures remains inconsistent.

**Methods:**

We investigated the association between DXA-measured LM and clinical bone fractures in community-dwelling elderly Japanese men from the Fujiwara-kyo Osteoporosis Risk in Men (FORMEN) cohort study. The source population consisted of 948 elderly men who underwent LM measurement by DXA at the time of the 2017–2019 survey. Among these, 780 participants who provided complete information regarding clinical fracture experience in the follow-up survey conducted in 2022–2023 were included in the analysis (mean age at the time of the 2017–2019 survey, 80.2 years; standard deviation, 3.8).

**Results:**

From the 2017–2019 survey to the 2022–2023 follow-up survey, 52 participants had experienced one or more clinical bone fractures including osteoporotic and non-osteoporotic fractures. Odds ratios of clinical bone fracture for arm LM, leg LM, and leg-to-trunk LM ratio were significantly lower than 1.0 after adjusting for potential confounding factors including age, nutrition intake, and physical performance.

**Conclusion:**

DXA-based appendicular LM predicts future clinical bone fractures in elderly Japanese men, suggesting that elderly men with low skeletal muscle mass, which can be accurately estimated from appendicular LM, may be prone to future clinical fractures. DXA-measured LM thus provides additional information useful for the management of bone fractures.

**Supplementary information:**

The online version contains supplementary material available at https://doi.org/10.1265/ehpm.25-00449.

## Background

Bone fracture is a global public health issue [1], and fracture-related burden is projected to increase over the coming decades [2]. Bone fractures pose a significant socioeconomic burden for both women and men in aging societies [3]. The number of hip fractures is estimated to approximately double to 2.6 million by 2025 and 4.5 million by 2050, with a greater percentage increase in men (310%) than in women (240%) [4]. In addition, the incidence of hip fracture has been reported to be higher in Caucasians than in Asians [3], and according to a systematic review and meta-analysis, there are racial and ethnic differences in the risk of fractures [5]. Meanwhile, sarcopenia is a progressive and generalized skeletal muscle disorder involving the accelerated loss of muscle mass and function [6]. A systematic review reported the pooled prevalence was 10%–16% of elderly people worldwide [7]. Other meta-analysis reported that sarcopenia was significantly associated with fracture in middle aged and elderly people [8].

Since skeletal muscle accounts for the largest proportion of lean mass (LM), LM is generally used to estimate skeletal muscle mass [9]. Therefore, LM measured by dual-energy X-ray absorptiometry (DXA) may be associated with bone fractures [10]. It has been reported that LM value obtained by DXA shows very good accuracy compared to that of computed tomography and magnetic resonance imaging [11]. Moreover, radiation doses associated with DXA are very low, and in fact, radiation dose per measurement (0.004 mSv) is much lower than natural background radiation per year (2.4 mSv) [12]. Furthermore, DXA can provide both whole-body and regional (arm/trunk/leg) LM [13], allowing also for the evaluation of body LM distribution [14].

However, evidence regarding the relationship between DXA-measured LM and fractures remains inconsistent. The Geneva Retirees Cohort study found that a low LM measured by DXA is an independent risk factor for incident fractures independently of the Fracture Risk Assessment Tool probability [15]. On the other hand, the Osteoporotic Fractures in Men Study cohort [10] and the Framingham Study Original and Offspring cohorts [16] found no association between low LM measured by DXA and fractures. In addition, no large-cohort study has investigated associations between DXA-measured LM and bone fractures in Asia. Therefore, detailed studies are needed to clarify how low skeletal muscle contributes to bone fractures. To this end, the present study aimed to examine the association between DXA-measured LM and clinical bone fractures in community-dwelling elderly Japanese men from the Fujiwara-kyo Osteoporosis Risk in Men (FORMEN) cohort study. We hypothesized that DXA-measured LM would be associated with clinical bone fractures independently of other confounding factors.

## Methods

### Study population

The FORMEN cohort study, a community-based, single-center, prospective cohort study aimed at determining risk factors for osteoporotic fractures, began in 2007–2008 in Nara Prefecture, Japan [17]. Follow-up surveys that included similar items as those of the baseline (2007–2008) survey, as well as items for identifying outcomes, were conducted in 2012–2013, 2017–2019, and 2022–2023 [3]. Inclusion criteria for the 2007–2008 survey were as follows: men aged ≥65 years who lived in their homes in four cities (Kashihara, Nara, Yamato-koriyama, and Kashiba) in Nara Prefecture and were able to walk without the assistance of another person, provide self-reported information, and understand and provide written informed consent [17]. At the 2007–2008 survey, 2174 men were recruited with the cooperation of local residents’ associations and elderly people’s clubs organized in each district of the cities studied [17]. Body composition measurements by DXA were conducted at the time of the 2017–2019 survey [14]. The source population of the present study consisted of 948 elderly men who underwent DXA-based body composition measurement in the 2017–2019 survey. Among these, the present study analyzed 780 participants who provided complete information on the incident of clinical bone fracture and potential confounding variables from the 2017–2019 survey through the follow-up surveys conducted in 2022–2023 (mean age in the 2017–2019 survey, 80.2 years; standard deviation, 3.8). Subgroup analysis excluded 40 participants who were unable to provide information on both smoking status and 10-meter walking time, and included 740 participants.

### Body composition measurements

Whole-body and regional (arm/trunk/leg) body composition measurements (bone mineral content, fat mass [FM], and LM) were performed using only one DXA scanner (QDR-4500A; Hologic Inc., Bedford, MA, USA) in an examination car in the 2017–2019 survey. One experienced radiologic technician performed all quality control steps for the DXA scanner and all measurements. Participants wearing light clothing were instructed to lay supine on the device table while whole-body DXA images were acquired. The supine whole-body images were used to identify the arms, trunk, and legs according to the standard method recommended by the manufacturer, with the following anatomical landmarks: a) a horizontal line just below the chin; b) a vertical line that bisects the shoulder joint; and c) a lower pelvic dividing line (two lines of an inverted triangle) that bisects both femoral necks [13]. Then whole-body and regional (arm/trunk/leg) FM and LM values were obtained. In addition, whole-body and regional FM and LM indices were calculated as FM and LM divided by height squared (kg/m^2^). Leg-to-trunk LM ratio (LTR) was calculated as leg LM divided by trunk LM.

### Anthropometry

In the 2017–2019 survey, participants had their weight and height measured in light clothing without footwear. Weight (kg) was measured using a bathroom scale. Height (cm) was measured using a height chart. Body mass index (kg/m^2^) was calculated as weight divided by the square of height. Waist circumference (cm) was measured at the navel level.

### Walking time measurement

Measurement of maximum walking speed (10-m walking time) was performed by a physical therapist in the 2017–2019 survey. The walking speed test consisted of a 2-m acceleration zone, a 10-m zone where walking speed was measured using an automated timing system, and a final 2-m deceleration zone, totaling 14 m. Participants were instructed to walk at their maximum speed without running or stopping.

### Nutrient and energy intake estimation

Dietary nutrient intake over the past month was estimated using the Food Frequency Questionnaire for Osteoporosis Prevention and Management (FFQPOP) at the time of the 2017–2019 survey [18]. Responses to the FFQPOP were jointly verified by a dietitian and participants. Amounts of nutrient and energy intake were estimated using the Standard Tables of Food Composition in Japan [19].

### Incidence of clinical bone fracture after the 2017–2019 survey

Information on clinical bone fractures was obtained in the 2022–2023 follow-up survey using mail questionnaires, followed by additional telephone surveys for non-responders and responders with missing data. To obtain accurate fracture information, the date of fracture, the skeletal site of fracture, the situation in which the fracture occurred, and the use of radiographs for forming the diagnosis of fracture by a physician were recorded for each incident of fracture [3]. The skeletal sites of fracture were classified as the head, neck, clavicle, rib, proximal humerus, distal forearm, phalanx, spine, pelvis or sacrum, hip, lower legs, ankle or heel, and toes. All incidents of both osteoporotic and non-osteoporotic fractures were used as the outcome of the present study due to the small number of incidents.

### Statistical analysis

All statistical analyses were performed with IBM SPSS statistics Version 26. P < 0.05 was considered statistically significant. Participants were divided into four equally sized groups (quartiles [Q] 1–4) based on DXA-measured LM. To compare participants with and without clinical bone fractures, the unpaired t-test, Fisher’s exact test, or the Mann–Whitney U test was used. Odds ratios for a 1Q increase for clinical bone fracture were calculated using multivariate logistic regression analysis after adjusting for potential confounding variables including age, energy, calcium, vitamin D, alcohol intake, and 10-m walking time. There were no smokers among those who had experienced a fracture; therefore, we removed smoking from the adjustment variables. The variable 10-m walking time may lie on the causal pathway between LM and clinical bone fractures, and therefore may be a mediator rather than a confounder of the relationship. Therefore, to demonstrate the robustness of the association, we performed an additional model without adjusting for 10-m walking time (Model 1 in Table 3 and Table 4). Odds ratios for clinical bone fracture in Q2–Q4 versus Q1 were also calculated using multivariate logistic regression analysis after adjusting for the potential confounding variables. It is proposed that stringent adjustments for P-value in multiple testing are not necessary for exploratory studies if findings are clearly presented as preliminary hypotheses to be validated by subsequent confirmatory studies [20]. Accordingly, the present study did not conduct multiple testing adjustments in the calculation of P-values for odds ratios in Q2–Q4 versus Q1.

## Results

Table 1 shows differences in characteristics between participants with and without clinical bone fractures. Since the 2017–2019 survey, 52 participants had experienced one or more clinical bone fractures of the head, clavicle, rib, proximal humerus, distal forearm, phalanx, spine, hip, lower legs, ankle or heel, and toes. Among them, 27 participants had experienced clinical bone fractures at typical osteoporotic fracture sites (e.g., hip, spine, distal forearm). Participants with clinical bone fractures had significantly lower height, weight, body mass index, whole-body LM, arm LM, leg LM, and LTR, and were significantly older compared to those without clinical bone fractures. On the other hand, no significant differences were observed between participants with and without clinical bone fractures in trunk LM, energy, calcium, vitamin D, and alcohol intake, or 10-m walking time. Table 2 shows the numbers of participants with and without clinical bone fractures stratified by quartiles (Q). From the lowest to highest Q of whole-body LM, arm LM, leg LM, leg LM index, and LTR, significant decreases in the number of participants who had experienced one or more clinical bone fractures were observed, while the dose-response pattern across quartiles was not always monotonic. Table 3 and Supplementary Table 1 show odds ratios for a 1Q increase in LM variables for clinical bone fracture. Odds ratios for a 1Q increase in arm LM, leg LM, and LTR for clinical bone fracture were significantly lower than 1.0 after adjusting for potential confounding factors (i.e., age, nutrition intake, physical performance). Table 4 shows odds ratios for clinical bone fracture in Q2–Q4 versus Q1. Odds ratios in Q3 of arm LM, Q3 of leg LM, and Q4 of LTR were significantly lower than 1.0 after adjusting for potential confounding factors.

**Table 1:** Characteristics of participants in the 2017–2019 survey.

	**Without fracture after survey**			**With fracture after survey**			**P-value**
	
	**N**	**Mean**	**SD**	**N**	**Mean**	**SD**	
Age (years)	728	80.1	3.8	52	81.4	3.9	0.020
Height (cm)	728	162.3	5.7	52	160.7	5.6	0.040
Weight (kg)	728	61.8	8.2	52	59.1	9.0	0.026
Body mass index (kg/m^2^)	728	23.4	2.7	52	22.9	3.1	0.175
Waist circumference (cm)	727	85.5	7.7	52	83.8	9.6	0.225
Whole-body FM (kg)	728	14.4	4.6	52	13.4	4.9	0.132
Whole-body FM index (kg/m^2^)	728	5.5	1.7	52	5.2	1.9	0.290
Whole-body LM (kg)	728	43.1	4.7	52	41.6	4.9	0.036
Arm LM (kg)	728	4.9	0.7	52	4.7	0.8	0.029
Trunk LM (kg)	728	21.2	2.4	52	20.7	2.5	0.148
Leg LM (kg)	728	13.5	1.8	52	12.9	1.7	0.016
Whole-body LM index (kg)	728	16.3	1.4	52	16.1	1.5	0.296
Arm LM index (kg/m^2^)	728	1.9	0.2	52	1.8	0.2	0.139
Trunk LM index (kg/m^2^)	728	8.0	0.7	52	8.0	0.8	0.823
Leg LM index (kg/m^2^)	728	5.1	0.6	52	5.0	0.5	0.108
LTR	728	0.6	0.0	52	0.6	0.0	0.040
Energy intake (kcal/day)	728	1709	296	52	1760	383	0.360
Calcium intake (mg/day)	728	524	149	52	552	245	0.421
Vitamin D intake (µg/day)	728	11.8	2.6	52	11.9	2.4	0.859
Alcohol intake (kcal/day)	728	85.0	128.8	52	88.6	174.0	0.852
10-m walking time (sec)	695	5.9	1.4	49	5.9	1.0	0.965
Current smoker	684			0			0.102
Non-current smoker	40			52			

N, number; SD, standard deviation; FM, fat mass; LM, lean mass; LTR, leg-t-trunk lean mass ratio.

Unpaired t-test or Fisher’s exact test was used to assess differences between participants with no fracture and those with fracture.

**Table 2:** Numbers of participants with and without fracture by quartile.

		**Without fracture**	**With Fracture**	**P-value**
Whole-body LM, Q1	IQR, 39.7–45.8 kg	174	21	0.005
Whole-body LM, Q2		181	14	
Whole-body LM, Q3		186	9	
Whole-body LM, Q4		187	8	

Arm LM, Q1	IQR, 4.4–5.3 kg	175	20	0.003
Arm LM, Q2		178	17	
Arm LM, Q3		188	7	
Arm LM, Q4		187	8	

Leg LM, Q1	IQR, 12.3–14.7 kg	173	22	0.002
Leg LM, Q2		181	14	
Leg LM, Q3		187	8	
Leg LM, Q4		187	8	

Whole-body LM index, Q1	IQR, 15.4–17.1 kg/m^2^	178	17	0.124
Whole-body LM index, Q2		183	12	
Whole-body LM index, Q3		180	15	
Whole-body LM index, Q4		187	8	

Arm LM index, Q1	IQR, 1.7–2.0 kg/m^2^	177	18	0.054
Arm LM index, Q2		182	13	
Arm LM index, Q3		182	13	
Arm LM index, Q4		187	8	

Leg LM index, Q1	IQR, 4.8–5.5 kg/m^2^	179	16	0.040
Leg LM index, Q2		176	19	
Leg LM index, Q3		187	8	
Leg LM index, Q4		186	9	

LTR, Q1	IQR, 0.6–0.7	177	18	0.040
LTR, Q2		181	14	
LTR, Q3		183	12	
LTR, Q4		187	8	

Q, quartile; LM, lean mass; IQR, interquartile range; LTR, leg-to-trunk lean mass ratio.

LM variables were transformed to quartile values.

P-values were calculated using the Mann-Whitney U test.

**Table 3:** Odds ratios for a one quartile increase in lean mass for fracture.

	**Model 1, N = 780**				**Model 2, N = 740**			

	**Adjusted odds ratios**			**P-value**	**Adjusted odds ratios**			**P-value**
	
		**95% confidence interval**				**95% confidence interval**		
Whole-body LM	0.744	0.538	1.030	0.075	0.770	0.550	1.077	0.126
Arm LM	0.723	0.534	0.977	0.035	0.717	0.525	0.979	0.036
Leg LM	0.691	0.503	0.949	0.023	0.718	0.517	0.997	0.048
Whole-body LM index	0.849	0.654	1.101	0.217	0.871	0.666	1.139	0.313
Arm LM index	0.801	0.617	1.040	0.095	0.807	0.617	1.056	0.119
Leg LM index	0.777	0.596	1.012	0.061	0.807	0.615	1.059	0.123
LTR	0.747	0.574	0.973	0.031	0.754	0.571	0.994	0.045

N, number; LM, lean mass; LTR, leg-to-trunk lean mass ratio.

LM variables were transformed to quartile values. Logistic regression analysis was used.

Model 1 adjusted for the following factors:

Whole-body LM and leg LM: age, energy, calcium, and vitamin D intake, and height.

Whole-body LM index, leg LM index, and LTR: age, energy, calcium, and vitamin D intake.

Model 2 adjusted for the following factors:

Whole-body LM and leg LM: age, energy, calcium, vitamin D, and alcohol intake, 10-m walking time, and height.

Whole-body LM index, leg LM index, and LTR: age, energy, calcium, vitamin D, and alcohol intake, and 10-m walking time.

**Table 4:** Odds ratios for fracture in each quartile of lean mass relative to lowest quartile (Q1).

	**Model 1, N = 780**				**Model 2, N = 740**			

	**Adjusted odds ratios**			**P-value**	**Adjusted odds ratios**			**P-value**
	
		**95% confidence interval**				**95% confidence interval**		
Whole-body LM, Q1	ref				ref			
Whole-body LM, Q2	0.701	0.336	1.464	0.345	0.709	0.332	1.517	0.376
Whole-body LM, Q3	0.471	0.193	1.149	0.098	0.466	0.184	1.183	0.108
Whole-body LM, Q4	0.452	0.164	1.244	0.124	0.519	0.184	1.463	0.215

Arm LM, Q1	ref				ref			
Arm LM, Q2	0.896	0.445	1.804	0.759	0.802	0.389	1.654	0.550
Arm LM, Q3	0.366	0.146	0.919	0.032	0.316	0.120	0.836	0.020
Arm LM, Q4	0.478	0.184	1.239	0.129	0.491	0.187	1.288	0.148

Leg LM, Q1	ref				ref			
Leg LM, Q2	0.627	0.302	1.300	0.209	0.577	0.266	1.252	0.164
Leg LM, Q3	0.349	0.143	0.853	0.021	0.374	0.151	0.926	0.034
Leg LM, Q4	0.402	0.150	1.074	0.069	0.443	0.161	1.217	0.114

Whole-body LM index, Q1	ref				ref			
Whole-body LM index, Q2	0.726	0.335	1.572	0.416	0.686	0.307	1.533	0.358
Whole-body LM index, Q3	0.906	0.436	1.880	0.790	0.970	0.454	2.072	0.937
Whole-body LM index, Q4	0.507	0.210	1.222	0.130	0.539	0.221	1.316	0.175

Arm LM index, Q1	ref				ref			
Arm LM index, Q2	0.728	0.344	1.537	0.404	0.715	0.329	1.553	0.397
Arm LM index, Q3	0.752	0.354	1.599	0.459	0.749	0.342	1.638	0.469
Arm LM index, Q4	0.458	0.193	1.090	0.078	0.476	0.198	1.147	0.098

Leg LM index, Q1	ref				ref			
Leg LM index, Q2	1.219	0.604	2.461	0.580	1.281	0.613	2.680	0.510
Leg LM index, Q3	0.463	0.193	1.115	0.086	0.524	0.213	1.289	0.159
Leg LM index, Q4	0.588	0.250	1.385	0.224	0.659	0.273	1.589	0.353

LTR, Q1	ref				ref			
LTR, Q2	0.724	0.346	1.516	0.392	0.668	0.306	1.458	0.311
LTR, Q3	0.620	0.286	1.342	0.225	0.623	0.279	1.390	0.248
LTR, Q4	0.390	0.163	0.932	0.034	0.394	0.160	0.968	0.042

Q, quartile; N, number; LM, lean mass; LTR, leg-to-trunk lean mass ratio.

LM variables were transformed to quartile values. Logistic regression analysis was used.

Model 1 adjusted for the following factors:

Whole-body LM and leg LM: age, energy, calcium, and vitamin D intake, and height.

Whole-body LM index, leg LM index, and LTR: age, energy, calcium, and vitamin D intake.

Model 2 adjusted for the following factors:

Whole-body LM and leg LM: age, energy, calcium, vitamin D, and alcohol intake, 10-m walking time, and height.

Whole-body LM index, leg LM index, and LTR: age, energy, calcium, vitamin D, and alcohol intake, and 10-m walking time.

## Discussion

This is the first study to report on the association between DXA-measured LM and future clinical bone fracture in a community-based, single-center, large cohort of Japanese elderly men. Arm LM, leg LM, and LTR, but not trunk LM, were significantly associated with future clinical bone fracture in the present results. These results are consistent with those of the Geneva Retirees Cohort study, which reported that low LM measured by DXA was an independent risk factor for low-trauma fractures [15]. In contrast, other previous studies reported no significant associations between DXA-measured LM and fractures [10, 16]. The inconsistent results in previous studies may be attributed to differences in study design. Whole-body skeletal muscle mass can be accurately estimated from DXA-measured appendicular LM, such as arm LM and leg LM, and appendicular LM, but not trunk LM, is used in the equation for estimating whole-body skeletal muscle mass [9]. Thus, the results of the present study suggest that elderly men with lower skeletal muscle mass may be more likely to experience future clinical bone fractures, and that DXA-measured LM may provide additional information useful for the management of clinical bone fractures in Japanese elderly men. On the other hand, in this study, absolute LM (kg) was significantly associated with clinical bone fractures in multivariate analyses adjusting for confounding factors such as height, whereas the LM index (LM divided by height squared (kg/m^2^)) showed no significant association with clinical bone fractures. One reason for the lack of a significant association between LM index and clinical bone fractures may be the limited sample size and statistical power, but residual confounding factors that cannot be completely eliminated may also contribute. To clarify whether this association should be interpreted as the effect of low LM itself or of low LM combined with other factors, further detailed research is needed.

Although the mechanism underlying the association between skeletal muscle mass and bone fractures was not explored in the present study, causal effects of low skeletal muscle mass on bone fractures may be explained partly by hormonal crosstalk between muscle and bone, i.e., osteokines and myokines affecting distant muscles and bones, respectively [21]. Myokines, proteins secreted by skeletal muscle cells, are widely involved in bone metabolism, influencing bone resorption and formation by interacting with factors related to bone cell secretion or influencing bone metabolic pathways [22]. Mechanical stress caused by muscle contraction also affects bones through various myokines in the crosstalk between muscle and bone [21]. Mechanical stress is a fundamental factor in the intricate processes governing the growth, development, morphological shaping, and maintenance of skeletal mass [23], and bone is highly adaptive to habitual mechanical loading, including stresses caused by muscle contraction and gravity [24]. Therefore, skeletal muscle mass loss can lead to a loss of bone strength and fracture. However, discussions regarding muscle-bone crosstalk and myokines remain speculative and are not directly supported by the findings of this study.

Another possible mechanism underlying the association between appendicular LM and bone fractures may be related to falls. Falls are common in older adults and can result in serious bone fractures [25]. A previous study in the FORMEN cohort revealed that LTR measured by DXA was inversely associated with falls in Japanese elderly men, and that the association of LTR with falls was independent of the association of physical performance with falls [14]. These results indicate that elderly men with a larger distribution of leg LM relative to trunk LM tended to have a lower risk of later falls [14]. This association might be explained by the position of the center of mass, and older adults with a mass distribution centered at the abdomen have been reported to be more prone to falling [26]. Therefore, reduced muscle mass in the legs may increase the risk of falls and fractures resulting from falls. However, the mechanism underlying the association between skeletal muscle mass and bone fracture remains unclear. Further detailed studies will be needed to clarify how low skeletal muscle contributes to clinical bone fractures.

The present study has some limitations worth noting. First, participants were not randomly selected. The participants comprised individuals who met the entry criteria of the FORMEN study. Thus, they consisted of relatively healthy, community-dwelling elderly men who volunteered to participate. The generalizability of our findings to frailer populations, institutionalized individuals, or women is limited. Older adults who volunteer and engage in more hours of volunteering have been found to report higher levels of well-being [27]. In addition, volunteers may enjoy good health and longevity because being useful to others instills a sense of being needed and valued [28]. Therefore, caution should be exercised in generalizing the results of the present study. Second, the follow-up period was short, and thus, the number of outcome events (fractures) was small. Therefore, non-osteoporotic fractures, in addition to osteoporotic fractures, were included in the present analysis. Thus, this approach results in significant heterogeneity in outcome definitions. The mechanisms underlying osteoporotic fractures (bone fragility) differ from those of traumatic fractures, which may be more closely related to falls and external forces. Therefore, it is unclear whether the observed associations reflect skeletal fragility, fall risk, or both. Further detailed research is needed to establish preventive strategies for osteoporotic fractures. Furthermore, the small number of events (52 clinical bone fractures) limits the statistical power and model stability. In the present study, the quartile-based analyses showed some inconsistencies (e.g., significant associations in Q3 but not always in Q4 in Table 4), which may reflect the limited power. It is well known that multiple logistic regression analysis requires at least 10 events (not 10 samples) per independent variable. However, the number of 10 events per variable is only a guideline. Therefore, in Tables 3 and 4, we used 5 to 6 independent variables in Model 1 and 7 to 8 independent variables in Model 2.

## Conclusion

The present community-based, large-scale cohort study revealed that DXA-based LM may predict future clinical bone fractures in elderly Japanese men, suggesting that elderly men with low skeletal muscle mass, which can be estimated from appendicular LM, may be prone to future clinical bone fractures. DXA-measured LM may thus provide additional information useful for the management of clinical bone fractures.

## Abbreviations

DXA: dual-energy X-ray absorptiometry
FFQPOP: Food Frequency Questionnaire for the Prevention and Management of Osteoporosis
FM: fat mass
FORMEN: Fujiwara-kyo Osteoporosis Risk in Men
IQR: interquartile range
LM: lean mass
LTR: leg-to-trunk lean mass ratio
N: number
Q: quartile
SD: standard deviation

## Supplementary information

**Additional file 1:** **Supplementary Table 1.** Odds ratios for fracture.

## Declarations

### Availability of data and materials

The data that support the findings of the present study are available on request from the corresponding author KK.
